# Genetic relatedness of *Staphylococcus aureus* isolates obtained from cystic fibrosis patients at a tertiary academic hospital in Pretoria, South Africa

**DOI:** 10.1038/s41598-018-30725-x

**Published:** 2018-08-15

**Authors:** T. Goolam Mahomed, M. M. Kock, R. Masekela, E. Hoosien, M. M. Ehlers

**Affiliations:** 1 Department of Medical Microbiology, Faculty of Health Sciences, Prinshof Campus, University of Pretoria, Pretoria, 0001 South Africa; 2 Tshwane Academic Division (TAD), National Health Laboratory Service, Pretoria, 0001 South Africa; 3 Department of Maternal and Child Health, School of Clinical Medicine, University of KwaZulu-Natal, Durban, 4041 South Africa; 4 Ampath Laboratory, Highveld Office Park, Centurion, 0157 South Africa

## Abstract

Cystic fibrosis (CF) is an inherited recessive disease that affects mucocillary clearance in the lung, allowing it to be colonised with bacteria such as *Staphylococcus aureus*. To survive in the CF lung *S. aureus* adapts both phenotypically and genotypically, through various mechanisms. In this study, multiple specimens were collected from the participants and were processed routinely and were additionally cultured in chromogenic media. Multiplex PCR assays were employed to detect methicillin resistance and selected virulence and quaternary ammonium compound (*qac*) genes. Genetic relatedness of the *S. aureus* was determined using *agr*, SCC*mec* and *spa* typing as well as pulsed field gel electrophoresis (PFGE) and multi-locus sequence typing (MLST). Thirty-three *S. aureus* isolates were isolated, of which 51% (17/33) were methicillin resistant *S. aureus* (MRSA). The virulence and *qac* genes were more prevalent in MRSA than the methicillin sensitive *S. aureus* (MSSA) isolates. The PFGE analysis showed nine distinct pulsotypes while MLST showed eight sequence types. All the STs detected in this study, except for ST508 have been previously isolated from CF patients according to the literature. This study showed a genetically diverse *S. aureus* population with a high prevalence of virulence genes among the MRSA isolates from the CF clinic.

## Introduction

Cystic fibrosis (CF) is an inherited disease, which affects one in 2000 Caucasians, one in 12000 people of mixed ancestry and one in 34000 Africans in South Africa^1^. This recessive disease is caused by mutations in the CFTR gene, a gene that encodes for a chloride channel protein, i.e. CF transmembrane conductance regulator (CFTR) protein^2^. As a result of mutations in the CFTR gene, the CFTR protein is dysfunctional, causing impaired mucocillary clearance^2^. The mucus layer in CF patients is thus without adequate defence systems and colonisation of the airways by pathogens (e.g. bacteria, fungi and viruses) often occurs^2,3^. *Staphylococcus aureus* is one of the most frequently reported bacterial pathogens to infect the CF lung, especially in young children^2,4^.

By undergoing phenotypic and genotypic adaptations, *S. aureus* is able to persist in the CF lung^5^. One such mechanism by which *S. aureus* adapts to the CF lung is through genomic rearrangements, with mobile genetic elements and phage mobilisation being key players^5^. This movement of genetic material may affect virulence traits by either interrupting the expression of virulence genes (e.g. β-haemolysin gene) or by equipping *S. aureus* with virulence genes, such as the Panton-Valentine leukocidin (PVL) toxin^5,6^.

The PVL toxin is one of the most widely studied virulence factors in *S. aureus* and studies have shown that it can cause inflammation in the lung (using rabbit models)^7–9^. However, observational studies reporting on the PVL toxin in CF patients found no association between the PVL toxin and clinical outcome and as such its role in CF is still unclear^7,10,11^. Another important virulence mechanism of *S. aureus*, especially in CF patients, is biofilm formation^5^. The *S. aureus* biofilm is multilayered and is often composed of glycocalyx forming part of a slime layer^12^. *Staphylococcus aureus* can form biofilms based on two conditions: (i) polysaccharide intracellular antigen (PIA)- dependent formation and (ii) PIA-independent formation^12^. Polysaccharide intracellular (PIA)- dependent formation is produced from the products of the *ica* locus^12^. The *ica* locus is induced by the staphylococcal respiratory response regulator protein (SrrAB) and can also be induced by glucose, osmolarity, temperature or antibiotics^12^. The *ica* locus can be controlled by the reversible inactivation of the insertion sequence *IS*256^12^. In PIA-independent biofilm formation, Protein A (Spa) is essential^12^. The virulence of *S. aureus* is aided by methicillin resistance; patients infected with MRSA strains have a lower lung function than patients infected with MSSA strains^7^. Methicillin resistance is conferred by the *mec*A gene, which can be found on a mobile genetic element known as the staphylococcal cassette chromosome *mec* (SCC*mec*) element^7^.

In South Africa, there is very limited data regarding both MSSA and MRSA isolated from the CF lung. Thus, the aim of this study was to determine the molecular characteristics and genetic relatedness of *S. aureus* isolates using multiplex PCR assays and genotyping methods, such as SCC*mec*, *spa* and *agr* typing, followed by pulsed field gel electrophoresis (PFGE) and multi-locus sequence typing (MLST) on selected isolates.

## Materials and Methods

This study was conducted at a CF clinic in Pretoria from October 2013 to May 2014, with ethical approval from the Faculty of Health Sciences Research Ethics Committee, University of Pretoria (343/2013). Informed consent (and assent, for participants over the age of seven) was obtained from each participant as well as from his/her parent/guardian (when under the age of eighteen).

As per routine, cough swabs and/or spontaneously expectorated sputum specimens were collected from participants and processed by the Diagnostic Laboratory of Tshwane Academic Division (TAD), National Health Laboratory Service (NHLS) using culture (mannitol salt agar for *S. aureus*) and the VITEK^®^2 automated system (bioMérieux, France) for identification. Additionally, a nasal swab [ESwab LQ Amies Pernasal Flocked Applicator (Copan, USA)] was collected from each participant and along with the remaining portions of the routine specimens were cultured on ChromID MRSA/Chrom ID *S. aureus* bi-plate (bioMérieux, France) and incubated (Digital Oven, Scientific Engineering (Pty) Ltd, Roodeport, South Africa) at 37 °C, up to 72 h to obtain pure colonies. The identification of all presumptive *S. aureus* isolates was confirmed using the MALDI Biotyper (Bruker Daltonics, USA).

Genomic DNA was extracted using the *ZR Fungal/Bacterial DNA Miniprep* (Zymo Research Corporation, USA) kit. Multiplex-polymerase chain reaction (M-PCR) assays were used to: (i) detect the prevalence of selected antibiotic resistance and virulence genes, such as *mec*A, responsible for methicillin-resistance; *ica*AB, involved in biofilm formation; the insertion sequence 256 (*IS*256), associated with control of the *ica* locus and biofilm formation and the *luk*S/F-PV genes, which encodes for PVL toxin, (ii) detect genes associated with quaternary ammonium resistance (*qac*) i.e. *qac*A/B, *qac*C, *qac*G, *qac*H and *qac*J genes, (iii) SCC*mec* type of the MRSA isolates and (iv) *agr* type of *S. aureus* isolates^13–17^. All multiplex PCR assays were performed using the Qiagen Multiplex PCR master mix (Qiagen, Germany) according to the manufacturer’s instructions (Supplementary Table S1). The VITEK^®^2 automated system (bioMérieux, France) was used to determine the antimicrobial susceptibility of the MRSA isolates, isolated from both routine analysis and chromogenic media (using M100 document of the CLSI guidelines for 2013/2014).

Using the *Sma*I enzyme and *S. aureus* subsp*. aureus* ATCC® 12600™ (as reference marker), PFGE of the *S. aureus* isolates was performed as previously described^18–20^. Electrophoresis was performed using the Rotaphor system (Biometra, Germany) on a 1.2% gel for 25 h (at 14 °C) at an angle of 120° with a field strength of 6 V/cm and an increasing pulse time (linear) from 5 sec to 40 sec. The gel was stained with 1 L ethidium bromide solution [250 µL of ethidium bromide (10 mg/mL stock (Sigma-Aldrich, USA)] digitally captured using an transilluminator (DigiDoc-It, UVP, LCC, USA) and stored. GelCompar II (Applied Maths, Belgium) was used to analyse the banding patterns and construct a dendrogram, showing the percentage of relatedness by means of the Dice coefficient and unweighted pair group method with arithmetic mean (UPGMA) methods. In this study, a similarity coefficient of ≥80% was used to assign pulsotypes to the *S. aureus* isolates^21^. A major pulsotype was defined as having more than five isolates within the cluster and a minor pulsotype was defined as less than five isolates within a cluster. *Spa* typing was performed as previously described using the *TaKaRa Taq*^TM^ (Clontech Laboratories Inc., Japan) according to the manufacturer’s instructions and the gel image was analysed as with PFGE (using a similarity coefficient of ≥80%)^22^.

Based on the PFGE and *spa* typing analysis, representative isolates were sent for MLST (twelve isolates) and *spa* sequencing (ten isolates), which were performed as previously described^23–25^. The assembled *spa* sequences were analysed using the DNA Gear Software (Open source software available at w3.ualg.pt/~hshah/DNAGear/) to assign *spa* types. Assembled MLST sequences for each locus were compared to the sequences in the *S. aureus* database (saureus.mlst.net) and each sequence was assigned an allelic number. The allelic profile was compared to the other profiles in the *S. aureus* database and a sequence type was generated.

## Results

A total of 19 participants were enrolled in this study from patients attending the CF clinic. The population was predominantly male and had a median age of seven (ranging from one to 40 years old). Eleven of 19 participants were colonised with *S. aureus*, with the number of isolates obtained per participant ranging from one to six. Five of the eleven participants (45%) tested positive for *S. aureus* from routine analysis and chromogenic media (two participants with MSSA only and three participants with both MRSA and MSSA) and six of the eleven participants (55%) were culture positive for *S. aureus* from chromogenic media only (one participant with MSSA only, one participant with both MRSA and MSSA and four participants with MRSA only).

**Figure 1:**
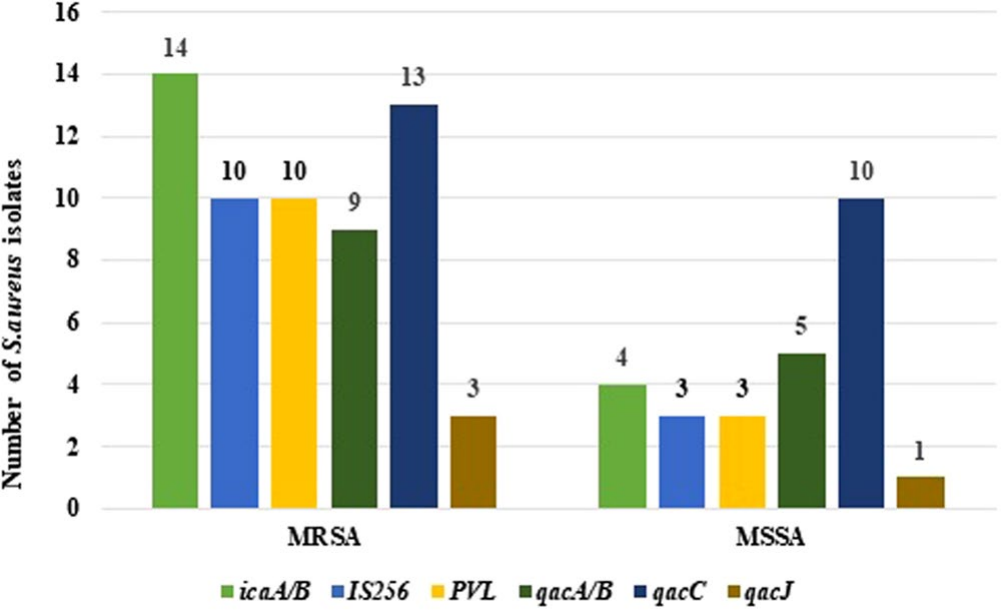
Bar graph comparing the prevalence of virulence and quaternary ammonium compound genes for MRSA and MSSA isolated from CF patients.

In total 33 *S. aureus* isolates were collected, ten from routine analysis and an additional 23 using the chromogenic media. All isolates were confirmed as *S. aureus* by MALDI-tof [MALDI Biotyper (Bruker Daltonics, Billerica, MA)]. The participants showed an almost equal distribution of MRSA and MSSA isolates, with 51% (17/33) being *mec*A positive. In both the MRSA and MSSA isolates, the *ica*A/B gene (biofilm formation) was most prevalent, followed by the *qac*C gene. No *qac*G and *qac*H genes were detected. Figure 1 shows the prevalence of the virulence and *qac* genes in the MRSA and MSSA isolates.

The SCC*mec* typing of the MRSA isolates showed that 53% (9/17) were SCC*mec* type I and 47% (8/17) were SCC*mec* type IV. No other SCC*mec* types were detected. The a*gr* type I was the dominant *agr* type.

The antibiotic susceptibility of the MRSA isolates was determined using the VITEK^®^2 automated system (bioMérieux, France). Two of the 17 MRSA isolates (12%) were susceptible to all antibiotics, 41% (7/17) were resistant to benzylpenicillin only, 23% (4/17) were resistant to two antibiotics (benzylpenicillin and another antibiotic) and 23% (4/17) were resistant to eleven antibiotics i.e. were multidrug resistant (Supplementary Table S2).

**Figure 2:**
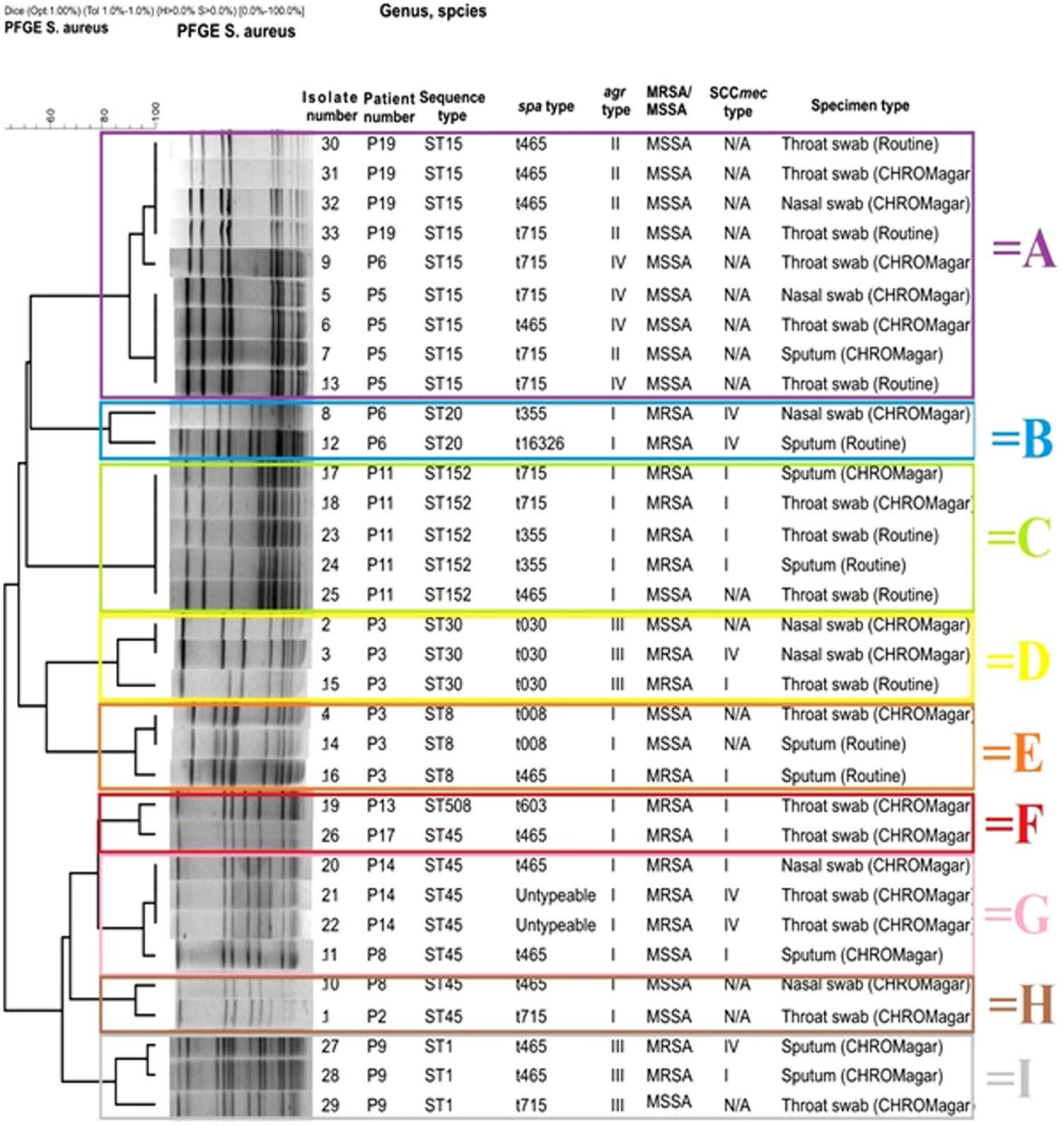
Pulsed field gel electrophoresis (PFGE) dendrogram of the *S. aureus* isolates showing the *spa* type, the *agr* type and the SCC*mec* type results. *Indicates isolates that have been sent for both MLST and *spa* sequencing and ~indicates isolates that have been sent only for MLST; the spa and ST indicated on the dendrogram were inferred based on these results.

Pulsed field gel electrophoresis showed two major pulsotypes (A and C) and seven minor pulsotypes (see Fig. 2). Two isolates (SA21 and SA22) were untypeable using *spa* typing, while the remaining isolates clustered into three groups with four outliers (Supplementary Fig. S2).

As can be seen in Fig. 2, two sequence types dominated, ST15 [27% (9/33)] and ST45 [21% (7/33)]. The distribution of the *spa* types of the ST15 strains (all MSSA) was as follows: (i) four isolates were t465 (three *agr* type II and one *agr* type IV) and (ii) five isolates were t715 (two *agr* type II and three *agr* type IV). The distribution of the *spa* types of the ST45 strains (all *agr* type I) was as follows: (i) four isolates were t465 (two SCC*mec* type I isolates, one SCC*mec* type IV isolate and one MSSA isolate), (ii) one isolate was t715 (MSSA) and (iii) two isolates were untypeable (SCC*mec* type IV).

## Discussion

The prevalence of *S. aureus* in this clinic was relatively high, occurring in 58% (11/19) of the participants. The prevalence of MRSA was 42% (8/19) and the prevalence of MSSA was 37% (7/19). In Europe and the USA, the prevalence of MSSA (15% to 56%) is usually higher than MRSA (2% to 23%)^7^. The prevalence of MSSA in this study was within the previously reported range; however, the prevalence of MRSA was much higher than the previously reported range and was also higher than the MSSA prevalence observed in this study. The reason for the higher prevalence of MRSA among these participants is unclear as the last reported prevalence of MRSA (2007) in this clinical setting in was 23%^26^. However, as participants attending the clinic only have biannually follow-up visits, the increased prevalence may possibly be due to the acquisition of MRSA circulating in the community.

The most prevalent virulence gene was the *ica*A/B gene [82% (14/17) of MRSA isolates and 25% (4/16) of MSSA isolates), which encodes for biofilm formation (polysaccharide-dependent). The prevalence of the *ica*A/B gene was higher in the MRSA isolates than the MSSA isolates, although biofilm formation has been known to occur in both MSSA and MRSA strains, especially in CF patients^7,12^. The prevalence of the *IS*256 gene was much lower than that of the *ica*A/B gene [59% (10/17) of the MRSA isolates and 19% (3/16) of the MSSA isolates]. The *IS*256 gene has been shown to be associated with biofilm formation (it controls the *ica* locus) and increased invasiveness, the lower prevalence of *IS*256 can be seen as a positive indicator of better outcomes for these patients^13^. The prevalence of the *luk*S/F-PV genes (encoding for the PVL toxin) were higher in the MRSA isolates than MSSA isolates, which is congruent with previous studies^7^. The PVL toxin has been associated with more severe disease in CF patients^27^.

The quaternary ammonium compound (QAC) resistance genes showed a high prevalence with the *qac*C gene being the most common gene detected in both MRSA [13/17 (76%)] and MSSA [10/16 (63%)] isolates. This higher prevalence of the *qac*C gene is unusual, typically the *qac*A/B gene is more prevalent (than the other *qac* genes) among the staphylococci^28^. However, the (low) prevalence of the *qac*G, *qac*H and *qac*J genes is expected as these genes are have a low prevalence in staphylococci^28^. Even though the *qac* genes are known to confer resistance to QACs, the resistance is found to be negligible if the QACs are used according to the manufacturer’s instructions^15^. However, the high prevalence of the *qac* genes is worrisome as these genes are associated with efflux pump mediated resistance and have the ability to confer resistance to antibiotics, such as aminoglycosides and β-lactams^15,28^. It has been observed that there is an association between biocide (QAC) resistance and antibiotic resistance^28^. According to the literature, a possible reason for this association is the close proximity of the *qac* genes and antibiotic resistance genes on mobile genetic elements, such as plasmids; in one instance the *qac*C gene was found on the same plasmid as the genes encoding for aminoglycoside (*aac*A-*aph*D), β-lactam (*bla*Z) and trimethoprim (*dfr*A) resistance in staphylocooci^28^.

Several of the *mec*A positive isolates showed an oxacillin-susceptible phenotype when the VITEK^®^2 automated system (bioMérieux, France) was used for antimicrobial susceptibility testing. Strains which show this phenomenon are referred to as oxacillin-susceptible MRSA (OS-MRSA) and are often overlooked during routine testing^29^.

The CF participants in this study showed a genetically diverse *S. aureus* population for both MRSA and MSSA isolates, with most participants being infected with more than one *S. aureus* strains. The collection and processing of the nasal swab together with the remainder of the sputum specimen and/or throat swab, using chromogenic media, from the participants may have contributed to the increased isolation of *S. aureus*, which may explain the isolation of multiple strains per participant. In most instances, these strains belonged to the same sequence type. However, for patient 3 (P3), two different sequence types were detected and clustered according to sampling location, ST30 was detected from the nasal swabs (upper airways) and ST8 was detected from sputum specimens (lower airways). Additionally, ST30 was detected from a throat swab, suggesting that colonisation of the upper airways may precede colonisation of the lower airways. Several studies have been conducted which compare the bacteria isolated from the upper and lower airways in CF patients^30–34^. These studies all suggested that the upper airways may act as reservoirs for the lower airways and the bacteria present in the upper airways may translocate to the lower airways through micro-aspiration or post-nasal drip^30–34^. The PFGE dendrogram (Fig. 2), showed two sequences types, ST15 [27% (9/33)] and ST45 [21% (7/33)], that predominated. Sequence type 15 (ST15) is rarely associated with MRSA and is found to be associated with MSSA, as was seen in this study where all ST15 isolates were MSSA^35^. Sequence type 45 on the other hand is associated with MRSA, which is similar to the findings of this study, where 71% (5/7) of the ST45 isolates were MRSA^36^. The SCC*mec* types of the ST45 MRSA isolates in this study were either type I or type IV. Both these strains have been detected previously with ST45-MRSA-I being detected sporadically in Hong Kong and ST45-MRSA-IV being an epidemic strain that is also known as the Berlin Epidemic Strain, WA MRSA-75 or USA600-MRSA-IV^36^. Additionally, this study showed a high prevalence of SCC*mec* type I (53%) in the MRSA isolates. Typically a low prevalence of SCC*mec* type I has been observed in countries such as the USA, however the results from this study correlated with a study conducted in Italy (2004/2005) which showed a prevalence of 49.5%^10,11,37,38^. This high prevalence of SCC*mec* type I, is of particular note in the South African context, where a study conducted in the same healthcare setting (in Pretoria) showed a prevalence of 3.1% and a study conducted on bacteraemia in South Africa did not detect SCC*mec* type I^39,40^.

The a*gr* type I was the most prevalent *agr* type [55% (18/33)] mostly associated with MRSA isolates [77% (13/17)]. Nastaly *et al*. (2010) had previously reported that 71% of all MRSA isolates belonged to *agr* type I, which is similar to the findings obtained in this study^41^.

The predominance of ST15 and ST45 in CF patients was not unique to this setting; in Europe and the Czech Republic these STs predominate as well^42,43^. Most of the STs identified in this study (ST1, ST8, ST20, ST30 and ST152), have previously been reported from CF patients in Europe^42,43^. However, ST508 (a single locus variant of ST45) has not been isolated from CF patients previously; this is the first report in CF patients. This sequence type (ST508) had been previously detected in Africa, mostly from nasal swabs^44^. However, in this study ST508 was detected from a throat swab.

While this study provided valuable insight into *S. aureus* in the CF lung, it did have several limitations. These limitations include that it was a single centre study (with a small number of participants) and only selected antibiotic resistance and virulence genes were detected.

## Conclusion

This study showed a high prevalence of important virulence factors, such as biofilm formation and the PVL toxin. These genes were much higher in the MRSA population than in the MSSA population, along with the genes for quaternary ammonium compound resistance. This clinic showed a genetically diverse *S. aureus* population, with ST15 and ST45 (epidemic strains) dominating.

## Electronic supplementary material

Supplementary Information
