# Supplementary material for: Genetic relatedness of Staphylococcus aureus isolates obtained from cystic fibrosis patients at a tertiary academic hospital in Pretoria, South Africa
Source: Sci Rep. 2018 Aug 15;8:12222. doi: 10.1038/s41598-018-30725-x (PMC6093922; doi:10.1038/s41598-018-30725-x)
Supplement: Supplementary file 1 — Supplementary Information [file 41598_2018_30725_MOESM1_ESM.pdf]

Title: Genetic relatedness of *Staphylococcus aureus* isolates obtained from cystic fibrosis patients at a tertiary academic hospital in Pretoria, South Africa (SREP-18-10024A)

Authors: T Goolam Mahomed[1], MM Kock[1,2], R Masekela[3], E Hoosien[4] and MM Ehlers[1,2]

Supplementary Information

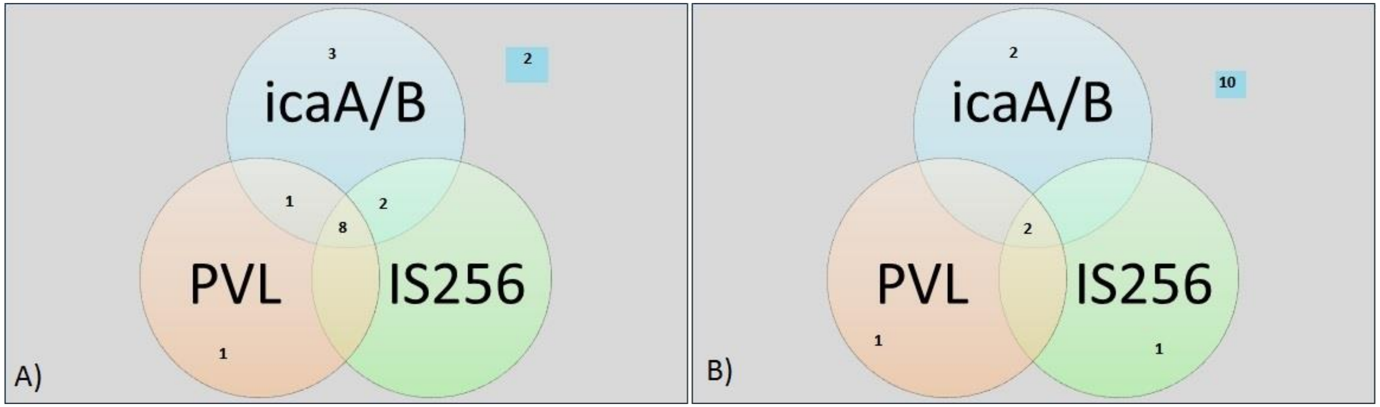

Figure S1: Distribution of the virulence genes in the MRSA (A) and MSSA (B) isolates. The Venn diagram shows the number of isolates with either the *ica*A/B genes, PVL toxin genes or IS256 genes and combinations thereof. The larger box encompasses the entire Venn diagram with the smaller box showing the number of isolates with none of the genes

Table S1: Nucleotide sequences of the primers and the amplification conditions used for the M-PCR assays for the detection of selected antibiotic and virulence genes, *agr* typing SCC*mec* typing, MLST, *spa* typing and *spa* sequencing of *Staphylococcus aureus* isolates

Target/Gene	Primer	Primer sequence (5'------------3')	Product size	Concentration	Reference	Cycling conditions
**Detection of antibiotic resistance and virulence genes**						
IS*256*	IS*256*-F	AGTCCTTTTACGGTACAATG	762 bp	0.4 µM	[13]	95ºC for 15 min; 94ºC for 30 sec, 57ºC for 90 sec and 72ºC for 90 sec for 35 cycles and at 72ºC for 10 min.
	IS*256*-R	TGTGCGCATCAGAAATAACG				
*ica*AB	*ica*-F	TTATC ATGCCGCAGTTGTC	546 bp	2 µM	[13]	
	ica-R	GTT TAA CGC GAG TGC GCT AT				
*luk*S/F-PV	*luk-PV*-F	ATCATTAGGTAAAATGTCTGGACATGATCCA	433 bp	0.1 µM	[14]	
	luk-PV-R	GCATCAAGTGTATTGGATAGCAAAAGC				
*mec*A	*mec*A-F	GTAGAAATGACTGAACGTCCGATAA	310 bp	1 µM	[14]	
	*mec*A-R	CCAATTCCACATTGTTTCGGTCTAA				
**Detection of quaternary ammonium compound resistance genes**						
*qac*A/B	*qac* A/B (F)	ATGCCTTATATTTATTTAATAATAGCC	321 bp	0.2 µM	[15]	95ºC for 15 min; 94ºC for 30 sec, 55ºC for 90 sec and 72ºC for 1 min for 35 cycles at 72ºC for 10 min
	qac A/B (R)	ATGCGATGTTCCGAAAATGTTTAAC				
*qac*C	*qac* C (F)	CTATGGCAATAGGAGATATGGTGT	417 bp	0.2 µM		
	qac C (R)	CCACTACAGATTCTTCAGCTACATG				
*qac*G	*qac* G (F)	TTTCGTTTGGAATTTGCTTT	203 bp	2 µM		
	qac G (R)	AATGGCTTTCTCCAAATACA				
*qac*H	*qac* H (F)	CAATAGTCAGTGAAGTAATAGGCAGTG	225 bp	2 µM		
	qac H (R)	TGTGATGATCCGAATGTGTTT				
*qac*J	*qac* J (F)	GGCCAACATTAGGCACACTTA	242 bp	0.2 µM		
	qac J (R)	TGACTTGATCCAAAAACGTTAAGA				
**SCC*mec* typing**						
SCC*mec* type I	1272F1	GCCACTCATAACATATGGAA	415 bp	0.08 µM	[16]	95ºC for 15 min; 94ºC for 30 sec, 58 ºC for 90 sec and 72ºC for 90 sec for 35 cycles and at 72ºC for 10 min
	1272R1	CATCCGAGTGAAACCCAAA				
SCC*mec* type II	β	ATTGCCTTGATAATAGCCYTCT	937 bp	0.2 µM		

Target/Gene	Primer	Primer sequence (5'------------3')	Product size	Concentration	Reference	Cycling conditions
	α3	TAAAGGCATCAATGCACAAACACT				
SCC*mec* type III	ccrCF	CGTCTATTACAAGATGTTAAGGATAAT	518 bp	0.25 µM		
	ccrCR	CCTTTATAGACTGGATTATTCAAAATAT				
SCC*mec* type IV	β	ATTGCCTTGATAATAGCCYTCT	937 bp	0.2 µM		
	α3	TAAAGGCATCAATGCACAAACACT				
	1272F1	GCCACTCATAACATATGGAA	415 bp	0.08 µM		
	1272R1	CATCCGAGTGAAACCCAAA				
SCC*mec* type V	ccrCF	CGTCTATTACAAGATGTTAAGGATAAT	518 bp	0.25 µM		
	ccrCR	CCTTTATAGACTGGATTATTCAAAATAT				
	5RmecA	TATACCAAACCCGACAACTAC	359 bp	0.1 µM		
	5R431	CGGCTACAGTGATAACATCC				
***agr* typing**						
*agr*B	pan-agr	ATGCACATGGTGCACATGC-	N/A	0.4 µM	[17]	95ºC for 3 min; 94ºC for 1 min, 55 ºC for 1 min and at 72ºC for 1 min for 25 cycles and 72ºC for 4 min
*agr*D	agr I	GTCACAAGTACTATAAGCTGCGAT	440 bp	0.4 µM		
*agr*C	agr II	GTATTACTAATTGAAAAGTGCCATAGC	572 bp	0.4 µM		
*agr*D	agr III	CTGTTGAAAAAGTCAACTAAAAGCTC	406 bp	0.4 µM		
*agr*C	agr IV	CGATAATGCCG TAATAC CCG	588 bp	0.4 µM		
**Multi-locus sequence typing**						
arcC	arcC –F	TTGATTCACCAGCGCGTATTGTC	456 bp	2 µM	[22]	95ºC for 5 min; 95ºC for 1 min, 55ºC for 1 min and 72ºC for 1 min for 30 cycles and 72ºC for 5 min
	arcC–R	AGG TAT CTG CTT CAA TCA GCG				
aroE	aroE-F	ATC GGA AAT CCT ATT TCA CAT TC	456 bp	2 µM		
	aroE-R	GGT GTT GTA TTA ATA ACG ATA TC				
glpF	glpF–F	CTA GGA ACT GCA ATC TTA ATC C	465 bp	2 µM		
	glpF–R	TGG TAA AAT CGC ATG TCC AAT TC				
gmk	gmk–F	ATC GTT TTA TCG GGA CCA TC	417 bp	2 µM		
	gmk-R	TCA TTA ACT ACA ACG TAA TCG TA				
pta	pta-F	GTT AAA ATC GTA TTA CCT GAA GG	474 bp	2 µM		
	pta-R	GAC CCT TTT GTT GAA AAG CTT AA				
tpi	tpi-F	TCG TTC ATT CTG AAC GTC GTG AA	402 bp	2 µM		
	tpi-R	TTTGCA CCT TCT AAC AAT TGT AC				
yqiL	yqiL-F	CAGCATACAGGACACCTATTGGC	516 bp	2 µM		
	yqiL-R-	CGTTGAGGAATCGATACTGGAAC				
***Spa* typing**						
	SPA1	GATTTTAGTATTGCAATACATAATTCG		0.32 µM	[23]	95ºC for 3 min; 94ºC for 30 sec, 55 ºC for 40 sec and at 72ºC for 50 sec for 35 cycles and 72ºC for 5 min
	SPA2	CCACCAAATACAGTTGTACCG		0.32 µM		
	SPA3	CTTTGGATGAAGCCGTTGCGTTG		0.32 µM		

Target/Gene	Primer	Primer sequence (5'------------3')	Product size	Concentration	Reference	Cycling conditions
			Spa sequencing			
	1095F	AGACGATCCTTCGGTGAGC		2 µM	[24]	95ºC for 15 min; 94ºC for 30 sec, 56 ºC for 30 sec and 72ºC for 1 min for 32 cycles and 72ºC for 7 min
	1517R	GCTTTTGCAATGTCATTTACTG		2 µM		

Table S2: Antimicrobial susceptibility profile of methicillin resistance *S. aureus* (MRSA) isolates

Patient	Isolate	Cefoxitin Screen	Benzylpenicillin	Oxacillin	Gentamicin	Ciprofloxacin	Moxifloxacin	Inducible Clindamycin resistance	Erythromycin	Clindamycin	Linezolid	Teicoplanin	Vancomycin	Tetracycline	Tigecycline	Fusidic acid	Mupirocin	Rifampicin	Trimethoprim/ Sulfomethoxazole	Notes
1	No *S. aureus*																			
2	1	MSSA isolate																		
3	2	MSSA isolate																		
	3	NEG	R	S	R	S	S	NEG	S	S	S	S	S	S	S	S		S	S	Resistant to Streptograms (SGA-SGB)<br>High level resistance to Mupirocin
	4	MSSA isolate																		
	14	MSSA isolate																		
	15	POS	R	R	R	S	S	NEG	R	R		R	R	R	S	R		R	R	VRSA*;<br>High level resistance to Mupirocin; oxalidionone resistant
	16	POS	R	R	R	S	S	NEG	R	R		R	R	R	S	R		R	R	
4	No *S. aureus*																			
5	5	MSSA isolate																		
	6	MSSA isolate																		
	7	MSSA isolate																		
	13	MSSA isolate																		
6	8	NEG	R	S	S	S	S	NEG	S	S	S	S	S	S	S	S	S	R	S	Resistant to Streptograms (SGA-SGB)
	9	MSSA isolate																		
	12	NEG	R	S	S	S	S	NEG	S	S	S	S	S	S	S	S	S	S	S	
7	No *S. aureus*																			

Patient	Isolate	Cefoxitin Screen	Benzylpenicillin	Oxacillin	Gentamicin	Ciprofloxacin	Moxifloxacin	Inducible Clindamycin resistance	Erythromycin	Clindamycin	Linezolid	Teicoplanin	Vancomycin	Tetracycline	Tigecycline	Fusidic acid	Mupirocin	Rifampicin	Trimethoprim/ Sulfomethoxazole	Notes
8	10	MSSA isolate																		
	11	NEG	R	S	S	I	S	NEG	S	S	S	S	S	S	S	S	S	S	S	Resistant to Streptogramins (SGA-SGB)
9	27	NEG	R	S	S	S	S	NEG	S	S	S	S	S	S	S	I		S	S	Resistant to Streptogramins (SGA-SGB) High level resistance to Mupirocin
	28	NEG	S	S	S	S	S	NEG	S	S	S	S	S	S	S	S	S	S	S	Resistant to Streptogramins (SGA-SGB)
	29	MSSA isolate																		
10	No *S. aureus*																			
11	17	NEG	R	S	S	S	S	NEG	S	S	S	S	S	S	S	S	S	S	S	Resistant to Streptogramins (SGA-SGB)
	18	NEG	R	S	S	S	S	NEG	S	S	S	S	S	S	S	S	S	S	S	
	23	POS	R	R	R	S	S	NEG	R	R	R	R	R	R	S	R		R	R	VRSA*; High level resistance to Mupirocin; oxalidionone resistant
	24	POS	R	R	R	S	S	NEG	R	R	R	R	R	R	S	R		R	R	
	25	MSSA isolate																		
12	No *S. aureus*																			
13	19	NEG	R	S	S	S	S	NEG	S	S	S	S	S	S	S	S	S	S	S	Resistant to Streptogramins (SGA-SGB)
14	20	NEG	R	S	S	S	S	NEG	S	S	S	S	S	S	S	S	S	S	S	
	21	NEG	R	S	S	S	S	NEG	S	S	S	S	S	S	S	S	S	S	S	
	22	NEG	R	S	S	S	S	NEG	S	S	S	S	S	S	S	S	S	S	S	
15	No *S. aureus*																			
16	No *S. aureus*																			
17	26	NEG	S	S	S	S	S	NEG	S	S	S	S	S	S	S	S	S	S	S	Resistant to Streptogramins (SGA-SGB)
18	No *S. aureus*																			
19	30	MSSA isolate																		
	31	MSSA isolate																		
	32	MSSA isolate																		
	33	MSSA isolate																		

***-All vancomycin resistant isolates were re-tested using the E-test and where found to be susceptible**

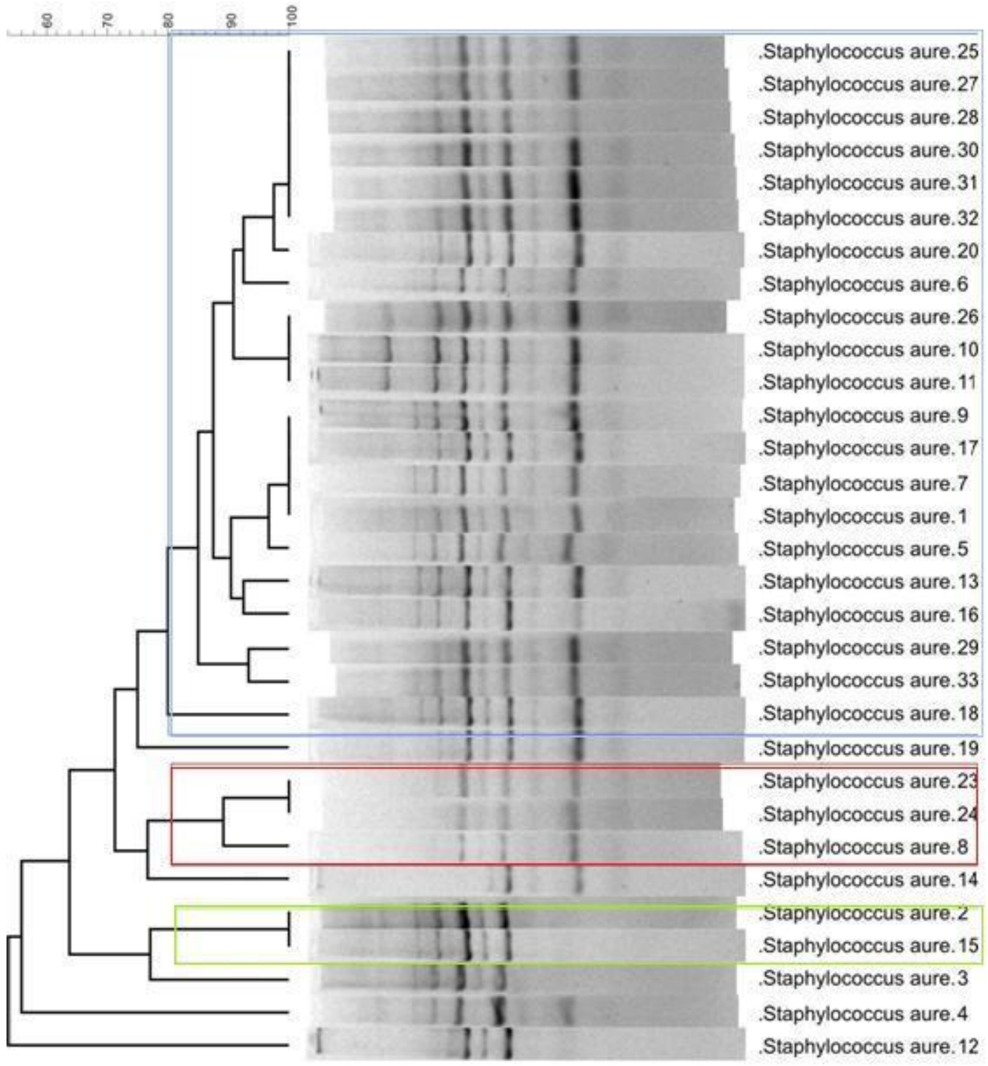

Figure S2: The spa typing dendrogram of the Staphylococcus aureus isolates. The dendrogram shows the three distinct groups and outliers of the typeable Staphylococcus aureus isolates using a similarity coefficient of 80%
